# Lipoprotein(a) and lipid profiles of patients awaiting coronary artery bypass graft; a cross sectional study

**DOI:** 10.1186/s12872-016-0393-1

**Published:** 2016-11-08

**Authors:** E. M. S. Bandara, S. Ekanayake, C. A. Wanigatunge, A. Kapuruge

**Affiliations:** 1 Department of Biochemistry, Faculty of Medical Sciences, University of Sri Jayewardenepura, Nugegoda, Sri Lanka; 2 Department of Pharmacology, Faculty of Medical Sciences, University of Sri Jayewardenepura, Nugegoda, Sri Lanka; 3 Cardiothoracic Unit, Sri Jayewardenepura General Hospital, Thalapathpitiya, Nugegoda, Sri Lanka

**Keywords:** Lipoprotein(a), Coronary artery disease (CAD), Lipid profile, Risk factors, Sri Lankan CAD patients

## Abstract

**Background:**

Lipoprotein(a) (Lp(a)) excess is an independent risk factor of coronary artery disease (CAD) and have shown wide ethnic variations. Further, lipid parameters used in the assessment and management of risk factors for CAD may not reflect accurately the disease or severity if the patients are on pharmacological interventions when compared to Lp(a). Lp(a) levels of Sri Lankan CAD patients awaiting coronary artery bypass graft are not documented.

**Methods:**

A cross sectional study was carried out with patients (*n* = 102) awaiting coronary artery bypass graft at a tertiary healthcare institution in Sri Lanka. Lp(a) was determined by immunoturbidimetric method (Konelab 20XT) and information on risk factors collected using a standardized questionnaire. The severity of CAD was determined by Gensini score. Lipid parameters and pharmacological treatment data were obtained from the Medical Records. Data were analysed using independent sample t-test, Pearson and Spearman tests respectively.

**Results:**

Total cholesterol (TC), LDL cholesterol (LDLc) and HDL cholesterol (HDLc) of the total study sample (average ± SD) were, 150 ± 36 mg/dL, 92 ± 36 mg/dL and 34 ± 9 mg/dL respectively with no significant difference irrespective of being on pharmacological treatment or not. All lipid parameters were significantly high (*p* < 0.05) in females. The average Lp(a) was 50 ± 38 (SD) mg/dL with no significant difference in males or females independent of being on treatment (50 ± 39 mg/dL) or not (49 ± 39 mg/dL) and above the cut off value (30 mg/dL).

**Conclusions:**

Despite pharmacological interventions 27 % of the study population had high LDLc and majority low HDLc. Mean Lp(a) was in excess irrespective of risk factors or being on treatment or not and is confirmed as an independent, potential marker for assessing the susceptibility for CAD especially in those with other intermediate risk factors but considered non-hyperlipidemic by conventional methods.

## Background

Cardio Vascular Disease (CVD) is a leading cause of death in the world. In Sri Lanka 40 % of proportional mortality is due to CVD [1]. South Asians are susceptible to Acute Myocardial Infarction (AMI) at an earlier age as they tend to develop higher risk-factor levels much earlier in life [2]. Sudden cardiac death (SCD) is estimated to account for 50 % of deaths from cardiovascular causes and about half of these deaths occur in subjects who were previously undiagnosed with heart disease [3, 4]. Coronary artery disease is the underlying cause in 80 % of SCDs, consequently, risk factors for coronary artery disease also predispose to SCD [3]. Risk factors for CVD comprise dyslipidemia, diabetes, hypertension, obesity, sedentary lifestyle, smoking, alcohol, family history, menopause and advancing age [1]. Further, homocysteine, fibrinogen, lipoprotein(a), low density lipoprotein particle size and c-reactive protein are the conditional risk factors that contribute to CVD [1]. Recently it has been shown that factors associated with culprit plaque rupture (CPR) to be different depending on clinical presentation. Hypertension was the only clinical predictor for STEMI, while advanced age, diabetes mellitus and hyperlipidaemia were the predictors in NSTEMI and unstable angina with no clinical predictor for stable angina [5].

The prevalence of hypercholesterolemia, hypertriglyceridemia and low HDLc were 14.86, 8.46 and 11.18 % among Sri Lankan adults [6]. A subsequent study reported elevated triglyceride (TG) and low HDLc in 12.2 % of subjects of <16 years and 24.5 % of subjects >16 years with a high prevalence in males [7]. A more recent study indicate an increasing trend in risk factors for CAD among Sri Lankans with the prevalence of hypercholesterolemia (>5.2 mmol/l), high LDLc (>3.4 mmol/L), high TG (>1.7 mmol/L) and low HDLc (<1.0 mmol/L) being 53.6, 24.7, 22.7 and 53.1 % respectively [8]. Statins are the most effective drug therapy used in controlling LDL cholesterol levels.

Management of lipid levels as part of risk factor modification associated with CAD is usually based on lipid profiles. Several studies indicate elevated Lp(a) is independently and linearly predictive of future adverse coronary events [9, 10]. Lp(a) excess increase the risk of premature CAD 3–100 fold depending on the absence or presence of concomitant risk factors [11, 12]. The inter-individual variability in the concentration of Lp (a) is mainly due to genetic regulation of rate of apoprotein (a) production [13]. Lp(a) promote pro-atherogenic processes by many mechanisms ie; interacting with fibrin and tissue matrix components in vessel walls [14, 15], inhibiting activation of plasminogen to plasmin [16], inhibiting plasmin mediated activation of transforming growth factor β (TGF-β) leading to increased proliferation of smooth muscle cells [17] and promoting inflammatory process by inducing monocyte chemotactic activity of vascular endothelial cells [18]. Thus Lp(a) would be a better risk marker for management of those with CAD and also for prediction of CAD susceptibility.

Patients with chronic stable angina undergoing diagnostic coronary angiography had significantly high (*p* = 0.002) plasma Lp(a) concentration than those without CAD. A significantly higher Lp(a) concentrations in CAD patients with a history of myocardial infarction (MI) (52.5 mg/dL) was observed compared to patients without MI (25.2 mg/dL) [19]. A South Indian study confirms the positive correlation with Lp(a) and severity of CHD [12]. As Lp(a) levels have shown wide ethnic variations [20] the present study assessed Lp(a) and lipid profile parameters of patients with confirmed diagnosis of CAD to observe the association of Lp(a) levels and lipid parameters and to study the effect of lipid lowering drugs on these parameters in a Sri Lankan study sample as these data are currently unavailable.

## Methods

### Study design and sample calculation

The current research was conducted as a prospective cross sectional study during the years 2013 and 2014. Study sample consisted of consenting patients (*n* = 102) awaiting Coronary Artery Bypass Graft (CABG) at the Cardiothoracic Unit, Sri Jayewardenepura General Hospital, Sri Lanka. The required sample size with an estimated prevalence of 30 % CVD [21], at 95 % significance level and a 10 % margin of error was 81. Since some diagnosed as CVD may not be CAD a study sample of 102 was enrolled.

### Lipoprotein(a) assay

Pre-operative blood samples were collected in to plain tubes. Serum was separated (3500 rpm, 5–10 min) and Lp(a) content was measured by immunoturbidimetric method (Thermo Scientific, Finland). Lipoprotein(a) calibrator and control (Thermo Scientific, Finland) were used for calibration. A specific anti serum (5 μL of anti-human Lp(a) from rabbit, NaN_3_ and NaCl) was added to buffered (140 μL; phosphate buffer saline) serum (24 μL), mixed and incubated at 37 °C. The absorbance of the immune-complex, produced from Lp(a) and anti-serum was measured at 340 nm (Konelab 20XT). The absorbance was considered proportional to the concentration of Lp(a).

### Lipid profile, risk factor data and severity of CAD

Data on total cholesterol (TC), low density lipoprotein cholesterol (LDLc), high density lipoprotein cholesterol (HDLc), triglyceride (TG) were collected from each patient’s data records and TC:HDLc ratio calculated. A standardized interviewer administered questionnaire was used to collect data on risk factors related to development of CAD. The Gensini score system was used to evaluate the severity of CAD from coronary angiography [22]. The data on coronary angiography were gathered from the medical records. The Gensini score was computed by assigning a severity score according to the degree of luminal narrowing and geographical importance of each coronary stenosis. The sum severity score of all coronary arteries was expressed as the Gensini score.

### Statistical analysis

The data are presented as mean ± 1 standard deviation (SD). *P* value of less than 0.05 (*p* < 0.05) was considered to be significant. Independent sample t-test and paired sample t-test were used for analysis of parametric variables. Correlations of parametric data were analysed by Spearman test.

## Results

Demographic data and risk factors of the study sample are included in Table 1. The study sample comprised of 102 patients awaiting Coronary Artery Bypass Graft (CABG) at Sri Jayewardenepura General Hospital, Sri Lanka. Males constituted 65.7 % (*n* = 67) of the sample. Majority of the patients (43.1 %) were between 51 and 60 years with 4.9 % less than 40 years. The average age of male and female patients was 56.9 ± 10 and 57.8 ± 7. From the study sample 70.6 % (*n* = 72), 53.9 % (*n* = 55) and 87.3 % (*n* = 89) individuals had a history of hypertension, diabetes mellitus and dyslipidemia respectively. Dyslipidemia was the most frequent disease related risk factor among both males and females. Family history of CHD was a risk among 54 % of the sample with females having a higher frequency (77 %).

**Table 1:** Demographic and risk factor data of the study sample

Variable		Total	Male (*n* = 67)	Female (*n* = 35)
Age		57 ± 9	56.9 ± 10	57.8 ± 7
Residency	Urban	59.8 %	55.2 %	68.6 %
	Rural	40.2 %	44.8 %	31.4 %
Monthly income (LKR)	<10 000	10.8 %	10.4 %	11.4 %
	11 000–30 000	72.5 %	70.1 %	77.1 %
	>31 000	16.7 %	19.4 %	11.5 %
Disease related risk factors				
Hypertension	Hypertensive	70.6 %	58.2 %	94.3 %
	Normotensive	29.4 %	41.8 %	5.7 %
Diabetes Mellitus	Diabetic	53.9 %	53.7 %	54.3 %
	Non Diabetic	46.1 %	46.3 %	45.7 %
Dyslipidemia	Dyslipidemic	87.3 %	83.6 %	94.3 %
	Non dyslipidemic	12.7 %	16.4 %	5.7 %
Family history of CAD	With family history	53.9 %	41 %	77 %
	No family history	46.1 %	59 %	23 %
Severity of CAD	Gensini score	48 ± 26	51 ± 25	44 ± 2

Lipid profile values (total cholesterol (TC), low density lipoprotein cholesterol (LDLc), high density lipoprotein cholesterol (HDLc), triglyceride (TG), TC:HDLc ratio), and (Lp(a)) of the study sample are stated in Table 2. The study sample consisted 87.3 % of patients on statins which comprised 83.6 % of males and 94.3 % females. The mean total cholesterol (<200 mg/dL) was well within the normal reference. LDLc of some females (36 %) on treatment were higher than recommended. Females on statins (*n* = 33) had significantly high TC (*p* = 0.001), LDLc (*p* = 0.02) and HDLc (*p* = 0.02) when compared to males on statins. From among 50 patients on statin therapy most patients (*n* = 48) were on atovastatin, either 20 mg (60 %) or 40 mg. However, the exact dose and the type of statin were not available for the other patients (*n* = 39). The TC:HDLc ratio was above 4.5 in majority of subjects.

**Table 2:** Lipid test measurements

Test (Reference range- mg/dL)	Total (*n* = 102)	Total (*n* = 102)		Male (*n* = 67)	Male (*n* = 67)		Female (*n* = 35)	Female (*n* = 35)	
		A (*n* = 89)	B (*n* = 13)		A (*n* = 56)	B (*n* = 11)		A (*n* = 33)	B (*n* = 2)
^a^TC (< 200)	150 ± 36	152 ± 36	137 ± 31	142 ± 29	143 ± 28	135 ± 33	167 ± 43^#^	168 ± 44*	151 ± 19
^a^LDLc (< 100)	92 ± 36	94 ± 37	82 ± 27	85 ± 24	87 ± 24	80 ± 28	105 ± 49^#^	105 ± 50*	98 ± 4.2
^a^HDLc (> 40)	34 ± 9	34 ± 10	31 ± 8	32 ± 9	32 ± 10	31 ± 8	37 ± 8^#^	37 ± 9*	33 ± 5.6
^a^TG (<150)	133 ± 60	134 ± 60	124 ± 64	127 ± 53	127 ± 51	129 ± 67	143 ± 71	146 ± 72	98 ± 45
^c^TC:HDL (< 5)	4.6 ± 1.2	4.6 ± 1.3	4.5 ± 1.0	4.6 ± 1.1	4.6 ± 1.2	4.5 ± 1.1	4.7 ± 1.4	4.6 ± 1.4	4.6 ± 0.2
^b^Lp(a) (< 30)	50 ± 38	50 ± 39	49 ± 39	47 ± 37	47 ± 36	49 ± 40	54 ± 41	54 ± 42	56 ± 41

*A;* on statins, *B;* without statins, *TC;* total cholesterol, *LDLc;* low density lipoprotein cholesterol, *HDLc;* high density lipoprotein cholesterol, *TG;* triglyceride

^#^Significant differences (*p* < 0.05) between male and female total concentrations; *Significant difference (*p* < 0.05) between male and females on statins

^a^Third Report of the National Cholesterol Education Programme (Adult Treatment Panel III), 2002 (23)

^b^Reference range as per lipoprotein(a) kit

Irrespective of statin therapy the Lp(a) concentration was not significantly different among males or females and was higher than 45 mg/dL. Variation of lipid profile values with high and low Lp(a) concentrations is stated in Table 3. The average TC and LDLc were within the normal range irrespective of Lp(a) being above 30 mg/dL (upper limit reagent kit) or more than 25 mg/dL as suggested cutoff for South Asians [12].

**Table 3:** Lipid profile results in patients with high (>30 mg/dL or >25 mg/dL) vs. low (<30 mg/dL or <25 mg/dL) Lp(a) levels

Lipid test	Lp(a) >30 mg/dL	Lp(a) <30 mg/dL	Lp(a) >25 mg/dL	Lp(a) <25 mg/dL
TC (< 200 mg/dL)	154.6 ± 32.2	143.3 ± 39.6	153.7 ± 34.3	142.0 ± 39.2
LDLc (< 100 mg/dL)	95.4 ± 28.9	84.4 ± 33.5	94.3 ± 30.1	83.8 ± 32.6
HDLc (> 40 mg/dL)	34.3 ± 7.4	33.0 ± 11.9	34.7 ± 9.8	31.7 ± 8.0
TG (<150)	128.7 ± 47.0	138.5 ± 77.0	131.2 ± 48.4	135.9 ± 82.6
TC:HDLc (< 5)	4.6 ± 1.2	4.6 ± 1.4	4.6 ± 1.2	4.6 ± 1.3

## Discussion

The mean concentrations of TC, LDLc, TG of both males and females on statin therapy and without statins were within the recommended [23] concentrations with lower HDLc. However, even though the therapeutic target of LDLc is <100 mg/dL for a person with CHD, 26.7 % (*n* = 27) from the total sample had higher LDLc concentrations and 8.8 % (*n* = 9) of females had TC concentration >200 mg/dL indicating inadequate control despite treatment. Haddad et al. [24] also reported mean values of LDLc as 146.7 ± 50.9 mg/dL and 118.9 ± 45.9 mg/dL of patients confirmed as CHD and that of controls respectively. The prevalence of hypercholesterolemia and mean total cholesterol concentration in Sri Lankan adults were 53.6 % and 5.35 ± 1.13 mmol/L (206.5 ± 43.6 mg/dL) respectively [8]. According to non-fasting results of total cholesterol, the prevalence of high TC was 20–25.9 % [25]. These data are a clear indication of the necessity in controlling not only total cholesterol but also the LDLc.

When considering the TG, 30.4 % males and females had triglyceride above 150 mg/dL. Correlating with above the waist to hip ratio of more than 90 % of the study sample indicated high risk [26]. HDLc concentrations of 77.5 % of patients were less than 40 mg/dL. The low HDLc correlated with the low physical activity level and the high prevalence of central obesity [26] in the present study sample. Majority of the patients [*n* = 89] in this study were on statins and mean concentrations of TC and LDLc were within the reference range [23]. However, since the exact dose and the statin are not available for some (*n* = 39) we are unable to make any inference on the dose and type correlations and lipid parameters. Notably, 45 % among the study sample was unaware that they were on statins and dyslipidemic despite good compliance. Thus the consumption of red meat, chicken, fish, egg with yolk and fast food was comparatively higher in the group who were not aware that they were dyslipidemic [26].

The above indicates that it is necessary to make the patients aware regarding usage of drugs which may reduce the adverse effects if any occur. Likewise, with prescription of drugs, information on lifestyle changes to maintain lipid parameters within normal ranges need to be imparted to these patients as above data shows this information is lacking.

TC:HDLc ratio of majority of patients was above 4.5. It clearly indicates that even though cholesterol was below the recommended value their HDLc need to be increased. Lifestyle changes are now being advocated to such patients. By and large a cut off of <5 for this ratio is being used in most Sri Lankan hospitals and thus it is necessary to evaluate the suitability of reducing the cut-off given the susceptibility of South Asians to Acute Myocardial Infarction (AMI) at an earlier age [2].

Lipoprotein(a) excess is an independent risk factor for coronary artery disease [9]. Lipoprotein (a) concentration of dyslipidemic (49.6 ± 38.5 mg/dL) and non dyslipidemic (51.1 ± 39.6 mg/dL) patients were not significantly different and 166 % higher than the cut-off (30 mg/dL) according to the present study and confirms its potential as an independent risk factor for CAD even among Sri Lankans. Despite the fact that male gender is a risk factor for CAD a non significant increase in Lp(a) in females was observed. This could be due to the lower samples size when compared to the males. A study where normolipidemic acute myocardial infarct (AMI) patients and controls of both Sri Lankan and Indian origin had been studied indicated a significantly high (*p* = 0.0001) concentration of Lp(a) [27] in the test group. This also clearly proves the importance of Lp(a) as an independent and an early marker for assessing the susceptibility for CAD especially in those with other intermediate risk factors such as hypertension, diabetes, overweight and obese with a family history but considered non-hyperlipidemic by conventional methods. However, around 2/3 of patients in this study samples had Lp(a) concentration >30 mg/dL, which is the upper limit of the reference suggested by the kit. This percentage is further increased when considering the cut-off value (>25 mg/dL) suggested for the South Indian population [12]. Except one patient who had a Lp(a) concentration of 1.3 mg/dL and was hyperlipidemic, all others had Lp(a) concentration >10 mg/dL. Therefore, a Lp(a) lower than 30 mg/dL is suggested as the cutoff to determine susceptibility of Sri Lankans to CAD considering the reported ethnic variation in Lp(a) [20]. However, for this Lp(a) levels of a control group needs to be studied.

Over 90 % of patients had TC within the recommended level and most had LDLc also within the normal range due to being on statin therapy. This indicates even with normal TC and LDLc, Lp(a) would indicate the CAD risk and can be considered as a potential independent risk marker as all the patients enrolled in this study were to undergo CABG.

High fat intake may lead to higher plasma Lp(a) levels in patients [28]. This could be correlated to the >20 % of expatriate workers both male and female who informed of dietary pattern changes towards high fat intake while living outside Sri Lanka. When their family history was considered, 64 % with family history had Lp(a) >30 mg/dL. This indicates that other factors such as diet may also have contributed to the increase in Lp(a) and the development of CAD in these patients.

Female sex, family history of CHD, high concentrations of TC and LDLc were reported to be associated with high concentration of Lp(a) [12]. In the present study an association with female sex and Lp(a) was observed. Europe and Atherosclerosis Society Consensus Panel also recommends screening of Lp(a) in individuals with intermediate or high risk of cardiovascular diseases/CHD. Lp(a) concentration of <50 mg/dL was suggested as a desirable level as a function of global cardiovascular risk [29]. In the present sample 2/3 of patients had Lp(a) of >30 mg/dL indicating a lower cutoff for Sri Lankans can also be considered as Lp (a) levels have shown wide ethnic variations [20] and are susceptible to change with the dietary patterns [28]. Similarly a Lp(a) concentration of 25 mg/dL had been suggested as the cutoff as risk of CHD in a South Indian study [12]. It is also recommended Lp(a) to be considered an additional marker for screening for CAD in susceptible individuals.

### Limitations

Comparison of data on Lp(a) and lipid profiles with an age and sex matched apparently healthy control group would have improved the study outcome.

A complete data set on the type and the intensity of statin therapy would have helped to find their effect on lipid parameters and the severity of the disease.

## Conclusions

Notwithstanding pharmacological interventions 27 % of the study population had high LDLc with more among females. Majority of patients had low HDLc and a TC:HDLc ratio of <5 which is within the recommended range. This highlights the urgent need for considering a lower cut-off for Sri Lankan population as there is clear evidence of increasing prevalence of CAD among Sri Lankans according to reported data. Lp(a) could be considered as an independent, potential marker for assessing the susceptibility for CAD in Sri Lankans especially in those with other intermediate risk factors but considered non-hyperlipidemic by conventional methods.

## Abbreviations

CABG: Coronary artery bypass graft
CAD: Coronary artery disease
CHD: Coronary heart disease
HDLc: High density lipoprotein cholesterol
LDLc: Low density lipoprotein cholesterol
Lp(a): Lipoprotein(a)
TC: Total cholesterol
TG: Triglyceride
